# Reversible splenial lesion syndrome associated with dengue fever: a case report

**DOI:** 10.1186/s13104-018-3491-6

**Published:** 2018-06-27

**Authors:** Pavithra Sathananthasarma, Praveen Nilendra Weeratunga, Thashi Chang

**Affiliations:** 1 Professorial Medical Unit, National Hospital of Sri Lanka, Colombo, Sri Lanka; 2 Department of Clinical Medicine, University of Colombo, Colombo, Sri Lanka

**Keywords:** Dengue, Splenium, Corpus callosum

## Abstract

**Background:**

Dengue virus infection in humans can lead to a wide range of clinical manifestations, from mild fever to potentially fatal dengue shock syndrome. The incidence of dengue fever is on the rise in tropical countries. Due to the increasing incidence of dengue fever worldwide, atypical manifestations of the disease are increasingly reported. In this article we report a patient with dengue haemorrhagic fever who presented with reversible splenial lesion syndrome.

**Case presentation:**

A 24-year-old Sri Lankan man who presented with fever and confusion was eventually diagnosed to have reversible splenial lesion syndrome based on brain imaging. Clinical, serological and haematological parameters confirmed a diagnosis of dengue haemorrhagic fever. His presentation, assessment, and management are described in this case report.

**Conclusion:**

Reversible splenial lesion syndrome is a condition which is radiologically characterized by reversible lesion in the splenium of the corpus callosum. It is associated with infectious and non-infectious aetiologies. This case report highlights the occurrence of reversible splenial lesion syndrome as a presenting feature of the expanding list of unusual neurological manifestations of dengue infection.

## Background

Dengue fever is a mosquito-borne viral infection caused by dengue virus, is endemic in Sri Lanka. The Epidemiology Unit of Sri Lanka reported 80,732 dengue fever cases, including 215 deaths during the first 6 months of 2017 [1]. Dengue virus infection in humans can lead to a wide range of clinical manifestations, from mild fever to potentially fatal dengue shock syndrome. Neurological manifestations of dengue fever are diverse and include encephalopathy, encephalitis, acute disseminated encephalomyelitis, Guillain–Barre syndrome, polyneuropathy, transverse myelitis and neuro-ophthalmic involvement [2, 3]. Here, we report a patient with dengue fever who presented with reversible splenial lesion syndrome. Due to the increasing incidence of dengue fever worldwide atypical manifestations of the disease are increasingly reported.

### Case presentation

A 24-year-old Sri Lankan man developed fever, profuse vomiting and diarrhoea followed by reduced level of consciousness over a 12-h duration. He had myalgia, arthralgia and frontal headache. He did not have photophobia, phonophobia, skin rash, fits, cough or urinary symptoms. He did not have any bleeding manifestations. He did not smoke tobacco or consume alcohol. There was no history of illicit drug abuse or high risk sexual behavior. He had been previously diagnosed with mild intermittent bronchial asthma.

On examination, he was febrile (101.3 °F), drowsy with a Glasgow coma scale of 11/15. There were no skin rashes or lymphadenopathy. No focal signs were noted in the neurological examination and fundoscopic examination was normal. His Pulse rate was 112 bpm; blood pressure was 100/60 mmHg with no postural hypotension; respiratory rate was 14/min. Rest of the general and systems examinations were normal.

His full blood count on admission showed white blood cells of 6 × 10^9^/L (Normal range [NR] 4.0–11.0 × 10^9^/L) with neutrophils of 59%; haemoglobin 14.3 g/dL (NR 13.5–16.5 g/dL) and platelet count of 74 × 10^9^/L (NR 150–450 × 10^9^/L). Erythrocyte sedimentation rate was 13 mm in first hour and C-reactive protein was 63 mg/l (NR < 5 mg/L). Serum electrolytes were normal with mild impaired renal function. Liver enzymes were elevated, alanine aminotransferase was 303 U/l (NR < 50 U/L) and aspartate aminotransferase was 482 U/l (NR < 50 U/L) with a total bilirubin of 10.6 µmol/L (NR 5–21 µmol/L). His urine analysis and coagulation profile were normal. Non-contrast CT brain demonstrated cerebral oedema. Lumbar puncture was precluded by a low platelet count.

Based on a working diagnosis of encephalitis, he was commenced on intravenous meropenem, vancomycin, aciclovir and dexamethasone, but were subsequently omitted once the diagnosis became evident.

**Fig. 1:**
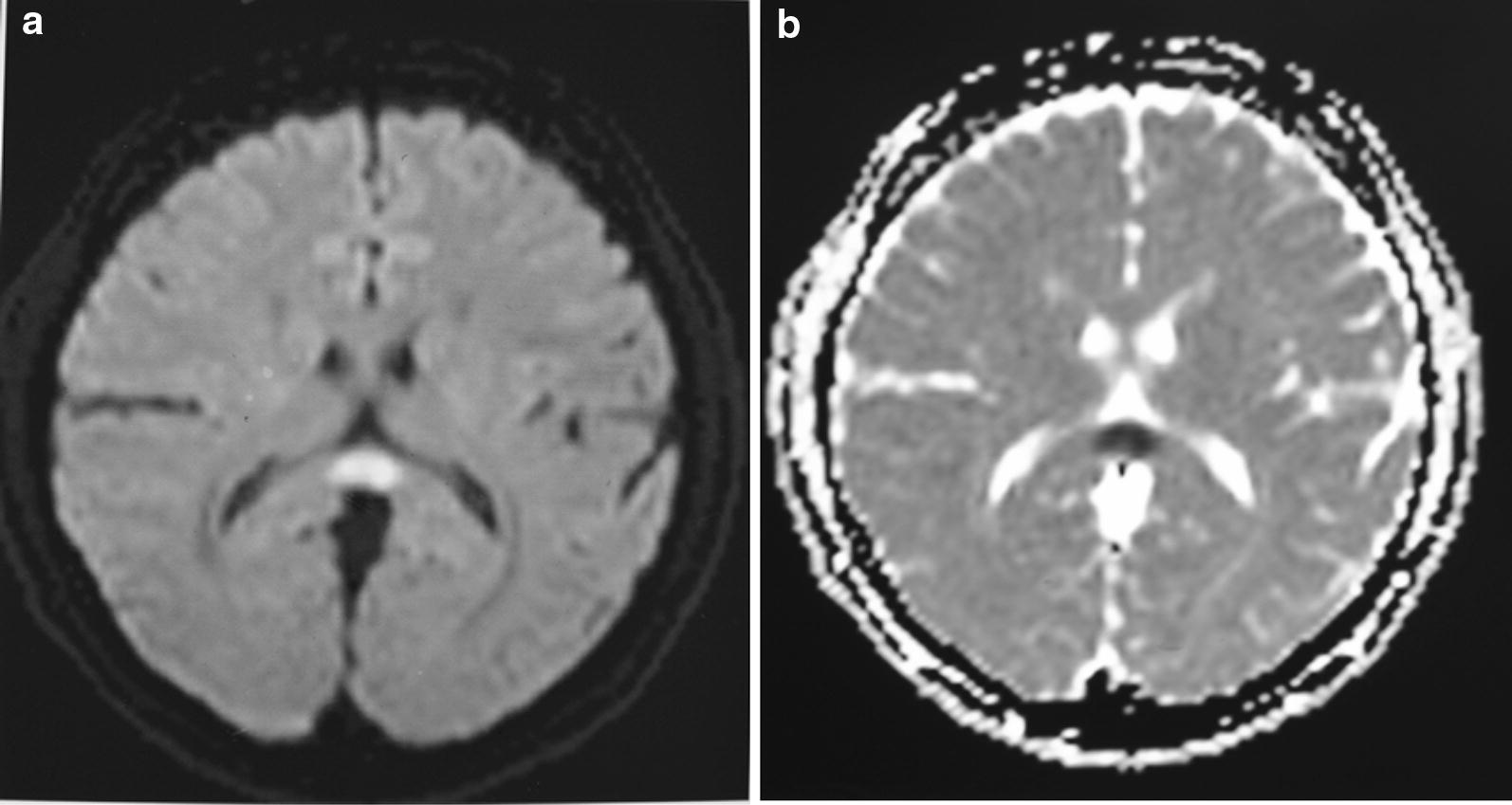
Initial MRI of brain (axial view) flair (**a**) and diffusion-weighted imaging (**b**) showing a lesion in the central splenium of corpus callosum

On further investigation, dengue IgM antibody in serum was positive on day 6 of fever. Blood and urine cultures did not yield any microbial growth. Electroencephalography showed generalized slow wave activity. The cranial MRI showed an isolated ovoid hyperintensity in the splenium of the corpus callosum with homogeneous hyperintense signal on diffusion-weighted imaging (DWI) (Fig. 1). A diagnosis of reversible splenial lesion syndrome (RESLES) was suspected based on the MRI appearance.

Standard dengue monitoring of vitals, urine output, haematocrit and platelet count were done. His platelet count dropped to a nadir of 31 × 10^9^/L, but subsequently increased. The white cell count steadily increased to 14 × 10^9^/L and then stabilized around 7 × 10^9^/L. Fluid leakage was not detected on repeated ultrasound scanning during the acute phase of illness.

**Fig. 2:**
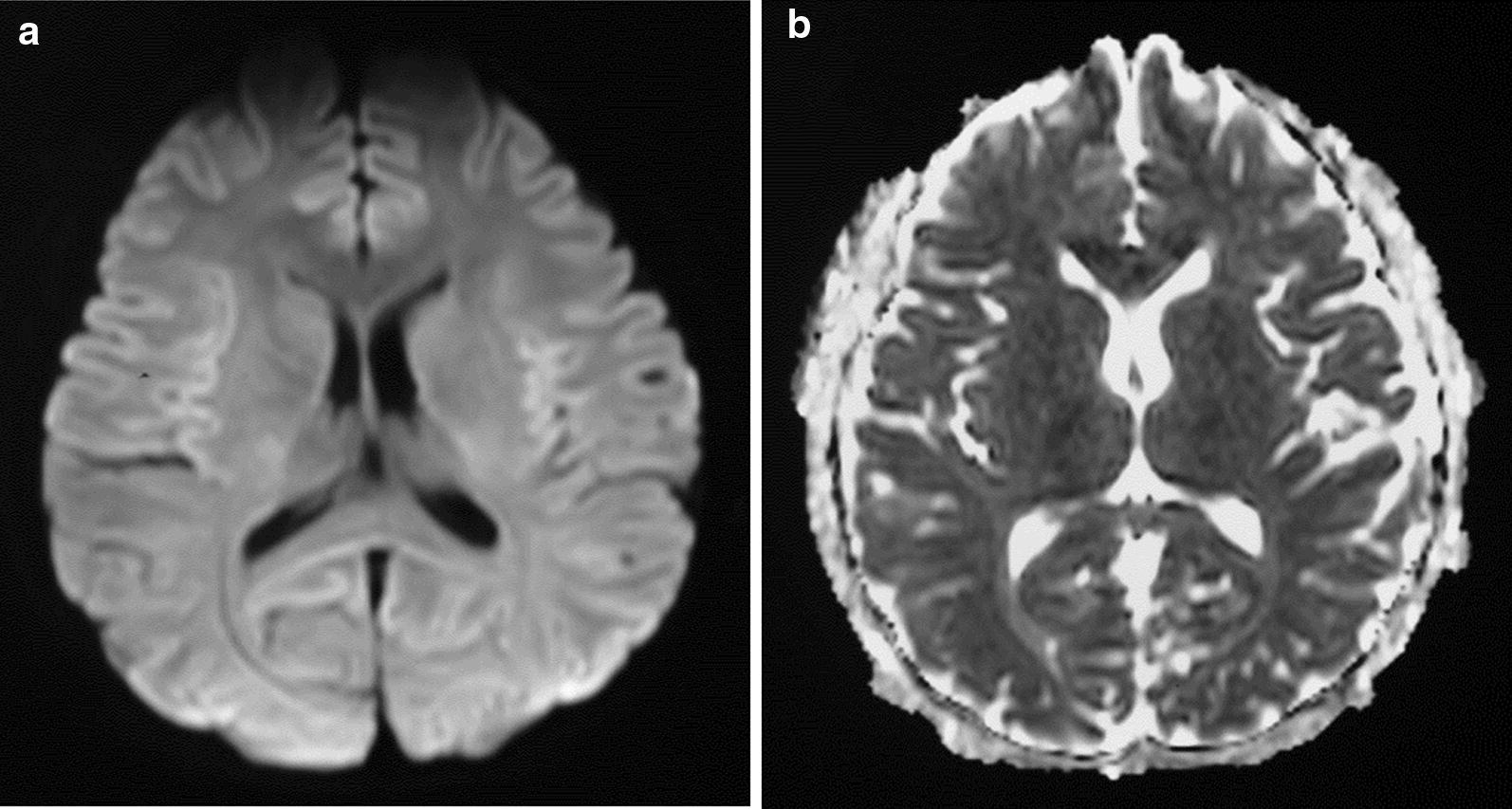
Follow-up MRI brain (**a**) and diffusion-weighted imaging (**b**) after 1 month showed resolution of the lesion

He was discharged from hospital on the tenth day of illness with complete clinical, heamatological and biochemical recovery. Follow–up cranial MRI done 1 month later showed complete resolution of the splenial lesion (Fig. 2).

## Discussion and conclusion

We report a case of reversible splenial lesion syndrome (RESLES) manifesting as the presenting feature in a patient with dengue haemorrhagic fever complicated by encephalopathy. The patient made a complete recovery while follow-up brain imaging revealed a complete resolution of the splenial lesion.

The corpus callosum is the largest fibre bundle that connects the cerebral cortex of the left and right cerebral hemispheres, and promotes the functional integration of sensory and motor functions. It is anatomically divided into rostrum, genu, body and splenium which play an integral role in relaying sensory, motor and cognitive information from homologous regions in the two cerebral hemispheres. Infarctions of the corpus callosum are not common because of the rich blood supply from three main arterial systems: the anterior communicating artery, the pericallosal artery, and the posterior pericallosal artery.

RESLES is a disorder radiologically characterized by reversible lesion in the splenium of the corpus callosum [3]. It is a non-specific radiological diagnosis associated with infectious and non-infectious aetiologies. In the case of infectious aetiologies, the syndrome is also called as mild encephalitis with reversible splenial lesion (MERS). Some authors name it as cytotoxic lesions of the corpus callosum (CLOCCs) based on its pathophysiology [4]. It is frequently observed in encephalitis caused by various pathogens such as influenza virus, rotavirus, measles, herpes virus, adenovirus, mumps, Epstein-Barr virus and Escherichia coli [3, 5–8]. Previously, only a single case of RESLES/MERS following dengue virus infection had been reported [9]. The patient described in the previous report was infected with dengue virus serotype 2, was younger than our patient and presented with delirium which progressed to ophthalmoplegia. However, the patient had demonstrated complete clinical and imaging resolution, consistent with the generally good prognosis associated with this rare clinico-radiological entity.

Among the non-infectious aetiologies, RESLES has been reported in association with anti-epileptic drug withdrawal, high-altitude cerebral edema (HACE), and metabolic disorders such as hypoglycaemia and hypernatremia [3, 5, 10, 11]. Lesions of the corpus callosum can cause a diagnostic dilemma, both for the radiologist and the clinician. Clinical presentation of RESLES is non-specific, associated with neuropsychiatric symptoms, mainly inter-hemispheric disconnection syndromes. Patients may experience gait disorders, apraxia, agraphia, tactile anomia and alien hand syndrome [5]. The diagnosis is based on the neuroimaging appearance of a non-enhancing, round-shaped lesion centered in the splenium of the corpus callosum that resolves after a variable duration. Although CLOCCs are non-specific with regard to the underlying cause, additional imaging findings and the clinical findings can aid in making a specific diagnosis. It is associated with a generally good prognosis except in those patients with an underlying severe disorder [3].

RESLES is mostly attributed to cytotoxic oedema with the exception of HACE in which vasogenic oedema is thought to be the underlying mechanism. The reason for the predilection for the splenium of the corpus callosum remains obscure, but a relative lack of adrenergic tone and failure of autoregulation at this site is one hypothesized mechanism [12].

Dengue infection is endemic in the tropics and subtropics causing periodic epidemics in this region. The identification of RESLES occurring in association with dengue infection expand the aetiological spectrum associated with it. The recognition of this distinct radiological appearance should prompt appropriate differential diagnosis and reassurance of a generally good prognosis provided that the underlying aetiology is appropriately managed.

## Abbreviations

RESLES: Reversible splenial lesion syndrome
ADEM: Acute disseminated encephalomyelitis
NR: Normal range
CRP: C—reactive protein
MRI: Magnetic resonance imaging
DWI: Diffusion weighted imaging
MERS: Mild encephalitis with reversible splenial lesion
HACE: High altitude cerebral oedema
